# Fractures Around the Knee—Significant Achievements During the Past 25 Years and Major Questions to Be Solved

**DOI:** 10.3390/jcm15093463

**Published:** 2026-05-01

**Authors:** Matthias Stockinger, Matthias Krause, Karl-Heinz Frosch

**Affiliations:** 1 Department of Trauma and Orthopaedic Surgery, University Medical Center Hamburg—Eppendorf, 20251 Hamburg, Germany; 2 Department of Trauma Surgery, Orthopaedics and Sports Traumatology, BG Hospital Hamburg, 21033 Hamburg, Germany

**Keywords:** distal femoral fractures, tibial plateau fractures, patella fractures, knee injuries, fracture fixation, minimal invasive surgical procedures

## Abstract

**Background**: Over the past 25 years, advances in knee surgery have been driven by an improved understanding of fracture morphology and associated injuries, as well as by significant technological progress. The introduction of novel classification systems has led to the refinement of treatment strategies, particularly with respect to the selection of surgical approaches. Furthermore, advances in biomechanical understanding have facilitated the development of new osteosyntheses designed to promote earlier rehabilitation while simultaneously reducing complication rates. **Research Question**: Which key milestones over the last 25 years have significantly influenced treatment strategies for knee joint fractures, with a perspective on unresolved issues? **Results**: Recent advances in fracture management, osteosynthesis, imaging techniques, and biomechanical research have substantially improved clinical outcomes, including a reduction in infection rates and improved postoperative results. The implementation of new classification systems has enabled more precise preoperative planning, allowing surgeons to define approaches that ensure adequate visualization of the articular surface while facilitating optimal positioning of the osteosynthesis. In terms of osteosynthesis, the introduction of locking plate technology has become widely established and supported by biomechanical evidence and has largely replaced traditional methods such as tension-band wiring of the patella. Despite these advances, fracture management in geriatric patients remains a considerable challenge, as compromised bone quality frequently limits the ability to achieve sufficiently load-stable osteosynthesis. Direct visualization of the articular surface is essential for adequate assessment and reduction of the affected articular segment. However, there is currently no consensus on which surgical approach or possible extension is most appropriate while simultaneously ensuring a low complication rate.

## 1. Background

Over the past quarter-century, the management of fractures around the knee has undergone substantial transformation, supported by advances in imaging diagnostics, surgical strategies, and implant technology. Complex fracture morphologies, particularly intraarticular tibial plateau fractures (TPF) and distal femur fractures (DFF), which were once associated with high complication rates and moderate postoperative functional outcomes, can now be treated using strategies available and derived from newly developed classification systems. In this context, diagnostic imaging has evolved from conventional radiography with supplementary projections to high-resolution, three-dimensional modalities. Today, the routine diagnostic repertoire includes not only conventional radiography and computed tomography (CT) with three-dimensional reconstructions, which enable precise fracture analysis and surgical planning, but also, when indicated, potentially necessary magnetic resonance imaging (MRI) for assessing associated meniscal and ligamentous injuries, which are detected in up to 93% of TPF [1]. In the intraoperative setting, imaging technologies, such as three-dimensional image intensifiers and arthroscopic assistance, may be used in selected surgical scenarios, both in minimally invasive procedures and in the management of complex TPF, to confirm fracture reduction and the position of the osteosynthesis [2,3]. These technologies ensure significantly more accurate reduction and secure placement of the osteosynthesis, particularly in highly complex fracture patterns [2,3].

DFF, including Hoffa fractures, as well as TPF and patellar fractures (PFs), share the common characteristic of involving the articular surface of the knee joint, where accurate anatomical reconstruction is essential for optimal functional outcomes. One of the persistent challenges in the management of these injuries is the limited ability to adequately visualize the joint surface intraoperatively, which complicates precise fracture reduction. Conventional intraoperative radiography alone often provides insufficient visualization of the joint surface, frequently leading to residual incongruity [4]. Consequently, open reduction with optimal exposure of the joint surface, particularly of key fragments, such as the posterolateral-central segment, is required in most cases to achieve accurate anatomical reduction and to improve long-term clinical outcomes [5]. Over time, new surgical approaches and refined classification systems have been developed to facilitate the selection of fracture-specific approaches adapted to the specific fracture morphology [6].

A major milestone in fracture management around the knee was the development of locking plate technology and cannulated screw systems, which allow for more reliable fixation in osteoporotic bone and comminuted fractures while reducing the risk of secondary loss of reduction. The biomechanical requirements for osteosynthesis vary substantially among fractures involving the knee joint and must therefore be carefully considered during surgical planning. DFF and TPF are predominantly subjected to axial loads, whereas PF are primarily exposed to tensile forces. This difference has been recognized for decades and led to the traditional use of tension-band wiring for PF. However, biomechanical studies have demonstrated that this technique does not reliably convert tensile forces into compressive forces at the fracture site [7].

Both DFF and TPF exhibit a two-peak distribution, affecting young, active patients sustaining high-energy trauma as well as elderly patients who typically sustain low-energy trauma. In contrast to the previously stable incidence of DFF and TPF over many years, recent studies have demonstrated a marked increase to 27.4 per 100.000 for DFF and up to 28.7 per 100.00 in TPF [8,9,10,11].

## 2. Tibial Plateau Fractures

The causes of injuries have shifted from high-energy events such as falls from great heights or traffic accidents toward outdoor risk sports, as well as generally increased physical activity into advanced age [9,12]. Even patients with severe fracture patterns have high expectations regarding postoperative outcomes, often underestimating the risk of developing post-traumatic osteoarthritis [13]. In view of the rising incidence of complex fractures, patients increasingly expect optimal functional outcomes and early initiation of functional rehabilitation [13]. This development has necessitated the establishment of modern, fracture-specific treatment concepts, which have been strongly supported by technological advances in both diagnostic imaging and implant technology.

With regard to diagnostics, remarkable progress has been achieved, ranging from the conventional X-ray with supplementary projections to CT and MRI and extending to intraoperative imaging modalities such as arthroscopic assistance and three-dimensional fluoroscopy [2].

## 3. Osteosynthesis

**Figure 1:**
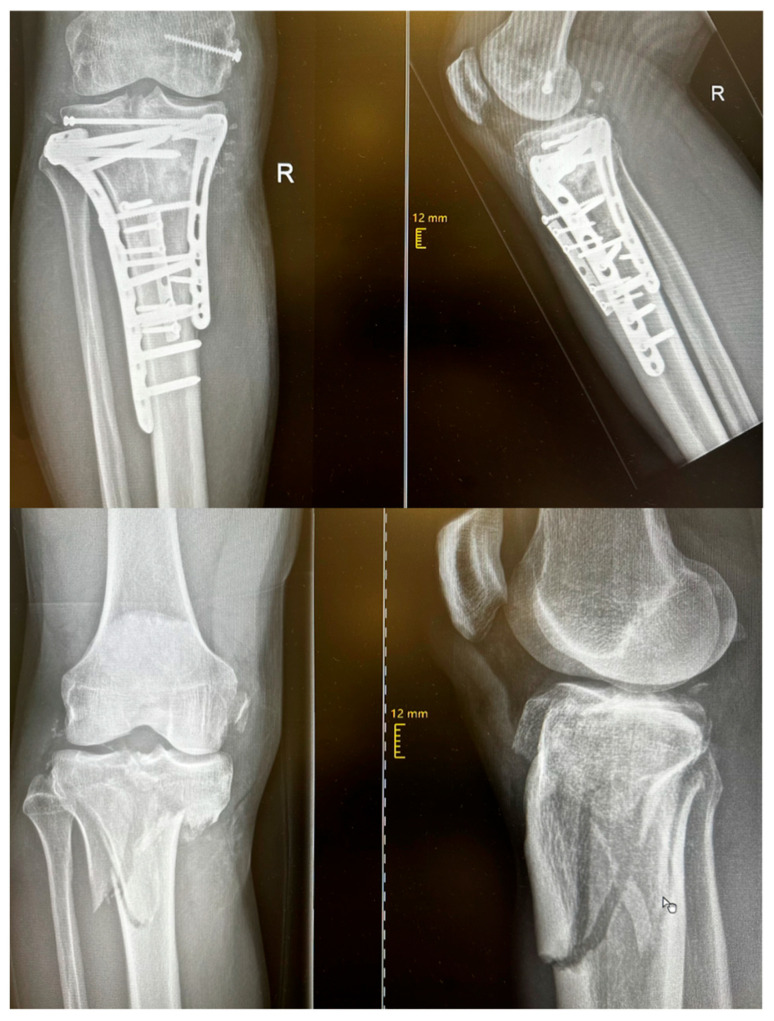
Radiographs of a 44-year-old patient with a tibial plateau fracture sustained during skiing. Following resolution of soft-tissue swelling, surgical treatment was performed via an anterolateral and a posteromedial approach. In addition, a bony avulsion of the medial collateral ligament (MCL) was refixed using screw osteosynthesis.

At the same time, innovative osteosynthesis systems have been established that meet the demands of complex fracture patterns. The development of locking plate systems, cannulated screws, and double-plate techniques applied to certain fracture patterns has proven biomechanically superior, as these approaches significantly reduce implant failure rates [14,15]. Locking plates provide angular stability and are therefore associated with lower rates of implant failure, allowing earlier weight-bearing on the affected limb [16,17]. When cannulated screws are used, guidewire placement enables precise screw positioning, which is particularly crucial for the fixation of key fragments. Figure 1 illustrates an example of surgical management of a complex bicondylar TPF treated with locking plates, a conventional plate, and cannulated screws. Minimally invasive treatments play a subordinate role and are reserved for non-displaced or minimally displaced fractures, typically involving screw osteosynthesis, sometimes with arthroscopic assistance. In particular, screw osteosynthesis using the jail technique has been shown to be superior to isolated screw fixation in fractures with mild depression, with respect to maximum load capacity and a reduced implant failure rate [18].

## 4. Arthroscopy/Fracturoscopy

With the substantial advancement of arthroscopic surgery, its indications have been expanded to include minimally displaced or non-displaced TPF [3]. Compared to open reduction and internal fixation (ORIF), arthroscopically assisted management of TPF is associated with shorter hospital stays and improved clinical outcomes while also offering superior options for the minimally invasive treatment of concomitant soft tissue injuries [19].

**Figure 2:**
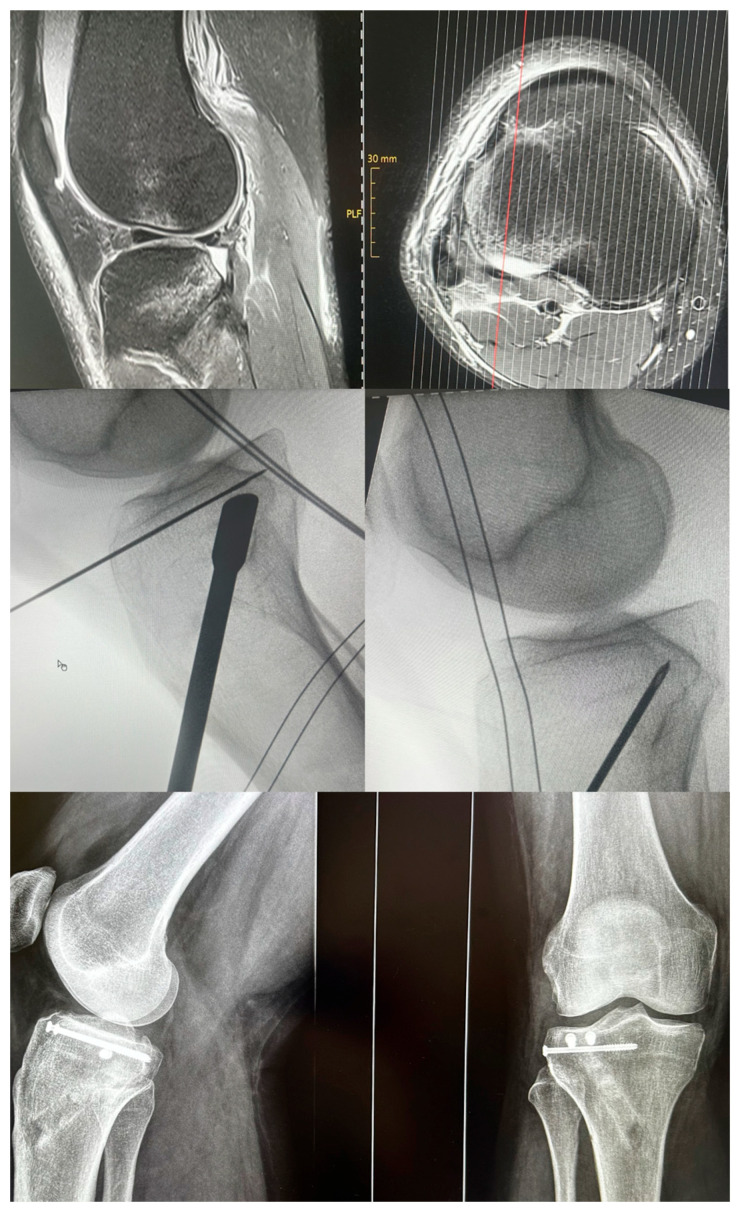
Preoperative MRI images and intraoperative radiographs of a female patient with an anterior cruciate ligament rupture and a posterolateral TPF. Shown is an example of surgical management using a minimally invasive, indirect reduction technique, screw osteosynthesis in a jail technique, and defect filling with allogeneic cancellous bone graft.

Figure 2 illustrates a minimally invasive closed reduction and internal fixation (CRIF) treatment of a posterolateral TPF, which was reduced under arthroscopic and fluoroscopic guidance. Minimally invasive fixation was achieved using cannulated screw osteosynthesis in a jail technique, with additional filling of the bone defect using allogeneic cancellous bone graft. In specific fracture patterns, such as bicondylar TPF, arthroscopy can provide additional information regarding fracture reduction and associated intra-articular injuries. However, this technique carries a potential risk of inducing an iatrogenic compartment syndrome. As an alternative approach, arthroscopy without continuous water pressure, referred to as fracturoscopy, can be used to visualize the articular surface. In this technique, the scope is introduced through the open surgical approach, allowing unobstructed fluid outflow and ensuring intermittent use of irrigation fluid when required [3].

## 5. Classification

There are numerous different classification systems; for TPF, more than 30 different systems have been described, with only moderate intra- and interobserver reliability [20], particularly among established classifications based on the two-dimensional assessment of anteroposterior fracture morphology (AO and Schatzker classification) [21]. The additional three-dimensional assessment of the axial plane using CT improves the understanding of fracture morphology, leads to improved surgical planning, and enhances the reliability of classification systems. To date, only five classification systems have been evaluated with respect to their inter- and intraobserver reliability, with simpler classifications, such as that proposed by Luo et al., demonstrating high reliability, though at the expense of providing less detailed information on fracture morphology [20]. The classification of Luo et al., together with subsequent refinements of classification systems, has led to new insights into the assessment of fracture morphology [22,23,24]. However, for surgical planning, the three-column model, which considers the extra-articular component of the fracture, as well as the 10-segment classification, has proven particularly useful [23]. A segment-specific assessment of the axial and articular fracture pattern, combined with knowledge of which parts of the tibial joint surface can be visualized through the different surgical approaches, supports a targeted selection of the most appropriate surgical approach [3].

TPF dislocations represent a particular challenge, as the severity of the associated osseous, soft-tissue, and neurovascular injuries makes accurate classification within existing systems difficult. In particular, in such complex combined injuries, the choice of surgical strategy is crucial to optimize postoperative outcomes, which frequently remain unsatisfactory [25,26].

## 6. New Approaches

Surgical management has increasingly moved away from the traditional concept of a single midline approach, which is associated with high complication rates and limited visualization of the articular surface, instead favoring an individualized, fracture-dependent surgical approach [27,28]. Crucial studies have shown that direct visualization of the fracture improves reduction accuracy. Through preoperative planning, appropriate surgical approaches with possible extensions, and the resulting improvement in fracture visualization have been key developments in modern treatment concepts [4]. In view of the frequent involvement of the posterolaterocentral segment (PLC), according to the ten-segment classification, reported in up to 85% of OTA/AO type C TPF, as well as the high rate of malreduction, particularly in the PLC segment, the application of the concept “direct approach to lateral tibial plateau fractures and stepwise extension as needed” has proven to be effective [4,5,23]. When an isolated anterolateral approach is used to treat fracture components extending into the PLC segment, malreduction rates of up to 89% have been reported [5,29]. Consequently, alternative and extended approaches have been developed with the aim of improving visualization of the lateral articular surface. Possible lateral extensions of the surgical approach are possible through osteotomy of the fibula, the femoral epicondyle, the Gerdy tubercle, and central subluxation of the lateral meniscus, which have proven effective in improving visualization [6,30,31,32]. In contrast to fibular head osteotomy, exposure of the common peroneal nerve is not required for lateral femoral epicondyle osteotomy, and the tight proximal tibiofibular joint does not have to be opened [33]. The lateral femoral epicondyle osteotomy is technically easier to perform, soft-tissue sparing, and allows for nearly complete visualization of the lateral tibial joint surface [33]. As opposed to fibular head osteotomy, which may be associated with complications such as secondary loss of reduction and implant migration, stable refixation of the osteotomy does not pose a technical challenge in lateral femoral epicondyle osteotomy, provided that the osteotomized bone block is of sufficient size, with a minimum of 2 × 2 × 1.5 cm [34].

However, it remains unclear which approach provides optimal visualization while ensuring low complication rates and allowing accurate positioning of the osteosynthesis. This question should remain the subject of future studies.

While comminuted fractures most frequently involve the lateral articular surface, the medial side is predominantly affected by posteromedial shear fractures [35]. Anatomical reduction in the extraarticular fracture consequently results in restoration of the articular surface. The fracture is then ideally stabilized using a posteromedial buttress plate through a posteromedial approach. However, due to the increasing prevalence of osteoporosis, multifragmentary fracture patterns are also increasingly observed on the medial side in up to 34% of cases [23]. In comminuted medial TPF, similar to the lateral side, the medial approach can also be extended by a medial femoral epicondyle osteotomy or an osteotomy of the tibial insertion of the superficial medial collateral ligament, which allows improved visualization of the articular surface [36].

In 1997, Lobenhoffer et al. introduced the posteromedial approach, which, partially modified, has been used for selected posterior comminuted TPF. While it provides direct access to the posterior tibial plateau, visualization and addressability of anterior fracture components remain limited, which may require intraoperative repositioning and further approaches [37,38].

## 7. Complications/Infection Rates

In the 1990s, infection rates of up to 87.5% were a major determinant of prognosis, particularly in bicondylar TPF [28]. With the introduction of staged surgical management, involving a five- to eight-day interval before definitive surgery, the use of fracture-specific surgical approaches and modern variable-angle locking plate systems, the average infection rate decreased to approximately 4.5% [28,39,40].

In addition, local application of antibiotics, such as vancomycin or antibiotic carriers, as well as the judicious use of a tourniquet, appear to further improve outcomes [41].

## 8. Distal Femoral Fractures

DFF primarily affects young males following high-energy trauma, with approximately 20% of cases being open fractures, and it is frequently associated with additional injuries such as solid organ injuries, chest trauma, and head injuries [42].

**Figure 3:**
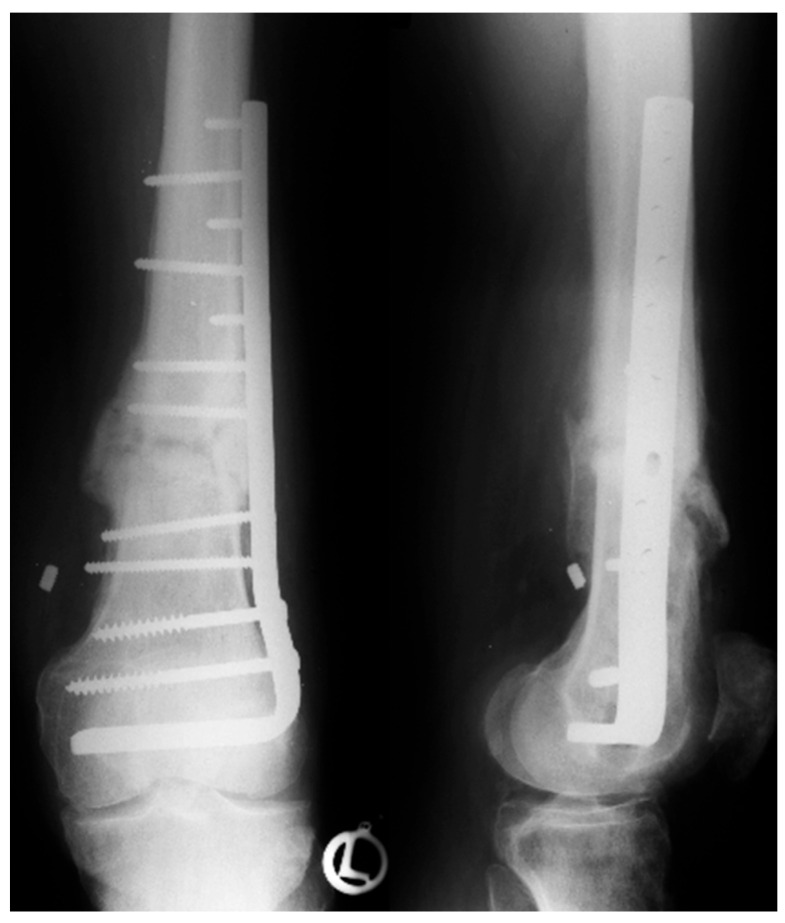
Illustrates the former use of a blade plate for the treatment of DFF. Reproduced with permission from Prof. Benedetto.

**Figure 4:**
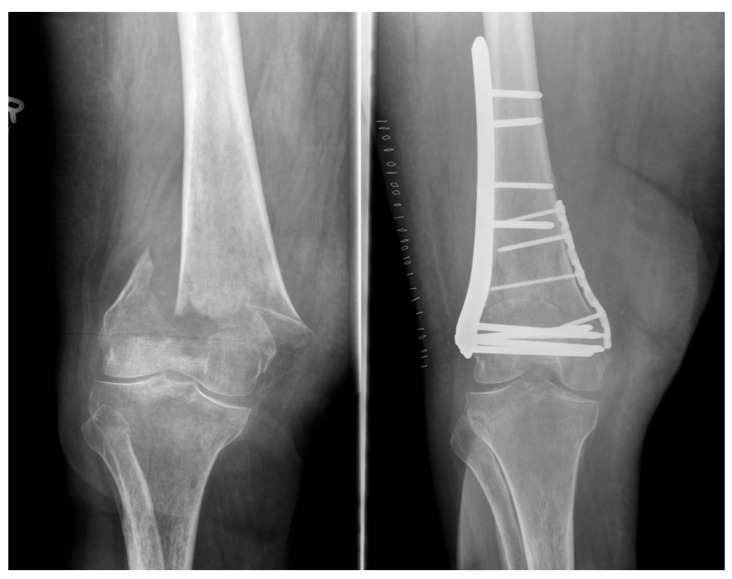
Radiographs of a 56-year-old female patient with an osteoporotic DFF treated with double-plating osteosynthesis.

However, elderly patients are also affected, typically sustaining DFF after low-energy trauma, such as falls. In this population, reduced bone quality and multiple comorbidities represent a challenge and are frequently associated with poor postoperative outcomes [43]. In addition, up to 5.5% of DFF are periprosthetic fractures, with the incidence increasing up to 30% in revision cases [44]. In contrast to the former use of dynamic compression plates and blade plates (Figure 3), the modern use of locking plates (Figure 4) allows for stable fixation and, depending on the fracture pattern, enables minimally invasive implantation [45]. Despite the availability of stable fixation options using locking plates, there are certain indications associated with a high risk of secondary loss of reduction, for which additional medial plating is recommended.

Patients with medial metaphyseal bone defects, low transcondylar or bicondylar fractures, poor bone quality, and pathological fractures particularly benefit from the use of this double-plating technique, as illustrated by the treatment example shown in Figure 4 [46,47]. A particular entity is the Hoffa fracture, a fracture of the lateral femoral condyle, less commonly of the medial condyle, in the coronal plane, which poses a surgical challenge and is often associated with poor long-term outcomes [48]. Especially in Hoffa fractures, the use of CT is crucial, as up to one-third of these fractures may be missed on conventional radiographs [49]. To date, the classification published by Letenneur et al. in 1978 is still widely used; it divides Hoffa fractures into three groups with further subclassification and can guide the selection of the surgical approach and osteosynthesis by considering fragment location, fragment size, and the fracture pattern [50,51]. Although the parapatellar approach is most commonly used, smaller Hoffa fragments in particular may not be adequately addressed; in such cases, the use of a posterior approach may be advisable [52]. Arthroscopically assisted treatment of Hoffa fractures is reserved for non-displaced or minimally displaced fracture types and offers the advantage of allowing identification of associated injuries that may not be detectable with open surgical approaches [53].

## 9. Patellar Fractures

Previous studies reported that PF predominantly affected men between 20 and 50 years of age, with 78.3% of cases resulting from traffic accidents and 13.7% from work-related accidents [54,55]. In contrast, more recent studies indicate that PF mainly affects elderly women and occurs in approximately 70% of cases following low-energy trauma [56,57]. In the context of demographic aging, periprosthetic PF has increased in recent years, with an incidence of approximately 2.5%, and now, together with poor bone quality, represents a growing operative challenge [57,58]. Operative strategies for PF have evolved over recent decades from metallic tension-band wiring to modern polymer band configurations and locking plate systems. This shift has been influenced by the substantial biomechanical demands placed on the extensor mechanism, the increasing complexity of multifragmentary fracture patterns, and the aim to reduce implant failure [59,60].

**Figure 5:**
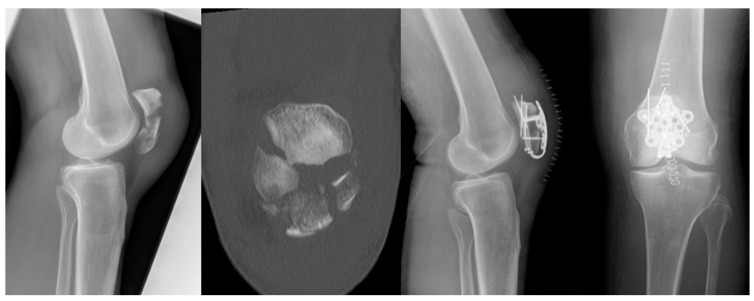
Radiographic and CT images of a 49-year-old female patient with a comminuted PF. Shown is an example of surgical treatment using patellar plate fixation with additional screw and K-wire osteosynthesis.

For many years, tension-band wiring with K-wires and metal cerclage represented the standard technique for PF. Theoretically, tension-band wiring should convert the high tensile forces generated by the extensor mechanism into compression at the fracture site. However, it has been demonstrated that this technique results in a static fixation, which is prone to secondary displacement when subjected to cyclic loading [7,61,62]. Recent biomechanical studies have shown that plate osteosynthesis provides high mechanical stability and is significantly superior to tension-band wiring osteosynthesis [63]. Modern plate designs with small screw diameter, varying sizes, and optional claw configuration allow different fracture patterns to be addressed and, where appropriate, enable plate osteosynthesis to be combined with other techniques such as screws or polymer bands. Figure 5 illustrates the surgical management of a comminuted PF involving the distal pole using a patellar plate and additional compression screws.

Special attention should be paid to fractures involving the distal pole of the patella, as these are associated with a high risk of secondary loss of reduction. Such fracture patterns are often difficult to diagnose accurately on conventional radiographs, particularly in the absence of primary displacement. A study published in 2013 demonstrated distal pole involvement in 88% of PF; however, this was identified on conventional radiographs in only 44% of cases [64]. Accordingly, CT is recommended to allow precise determination of fracture morphology and has been shown to result in modification of the operative strategy in up to 49% of cases [64,65].

## 10. Fibula

Proximal fibular fractures, when occurring in combination with Schatzker-type VI TPF, have a decisive impact on postoperative prognosis [66]. In addition, an intact proximal fibula appears to contribute to the stability of the lateral tibial plateau, allowing safe early weight-bearing mobilization and thereby facilitating faster patient recovery [67].

## 11. Bone Graft Substitutes/Biologics

Up until the early 2000s, autologous bone graft harvested from the iliac crest was considered the gold standard for filling bone voids in TPF, whereas bone allografts had already been used for years for acetabular reconstruction without long-term complications [68]. To fill large defects while avoiding donor-site morbidity, various bone substitute materials have been developed, with injectable calcium phosphate cements showing good short-term results in TPF [69,70]. However, possible disadvantages include slow resorption and delayed and unpredictable replacement, which may compromise fracture healing [71]. Further clinical evidence emerged from studies beginning in the late 1990s, showing that cancellous allograft can provide reliable mechanical support in TPF as well as a well-osteoconductive scaffold, while avoiding donor-site morbidity [72,73,74]. With the introduction of locking plate technology, impacted allogeneic bone grafts became a routine adjunct to support depressed articular fracture fragments [75]. Consequently, allogeneic bone grafts have evolved from an adjunct reserved for selected cases into an established component of reconstructive strategies in TPF, without donor-site morbidity.

Biologics such as platelet-rich plasma (PRP) have shown heterogeneous clinical results in fracture care around the knee. Promising results have been demonstrated in preclinical studies and in the treatment of non-unions; however, the benefits in acute fractures remain inconsistent [76].

## 12. Conclusions

The management of fractures around the knee has evolved substantially over recent years, reflecting continuous progress in medicine across areas such as biomechanics, fracture morphologies, implant technology, and imaging. These advances have led to improved osteosynthesis stability, reduced complication rates, and overall better functional outcomes.

One of the most transformative advances has been the routine integration of CT into preoperative planning. Compared with conventional radiographs, CT imaging enables a far more precise assessment of complex fracture morphology, particularly in TPF and DFF [2]. This improved understanding has facilitated the development of more detailed classification systems, such as the 10-segment classification, which allow surgeons to better identify relevant fracture components and determine fracture-specific surgical approaches [23]. Consequently, surgical planning has shifted from standardized midline approaches in TPF toward individualized strategies based on fracture morphology, associated soft tissue injuries, and the location of key fragments [27,28].

An important development has been the refinement of surgical approaches aimed at improving visualization of the articular surface [4]. Accurate anatomical reduction in intra-articular fractures remain a prerequisite for favorable long-term outcomes. Traditional approaches in TPF, such as the midline incision, have increasingly been replaced by fracture-specific approaches and stepwise approach extensions, allowing direct visualization of key fracture fragments while minimizing soft-tissue damage. This concept is particularly relevant in complex TPF involving the posterolateral-central segment, where insufficient exposure has historically been associated with high rates of malreduction [5,29]. New surgical approaches, including lateral extensions through osteotomy of the fibula of the femoral epicondyle of the Gerdy tubercle, as well as central subluxation of the lateral meniscus and modified posteromedial approaches, have therefore been developed to address these limitations. Which surgical approach extension provides the greatest advantages while minimizing morbidity has not yet been definitively clarified.

In parallel with improvements in diagnostic evaluation, major progress has been achieved in implant technology and biomechanical understanding. The introduction of angularly stable locking plate systems represents one of the most influential milestones in fracture management around the knee. Locking plates provide improved fixation stability, particularly in osteoporotic bone and in comminuted periarticular fracture patterns. Biomechanical studies have demonstrated that these implants reduce the risk of secondary loss of reduction and implant failure, thereby allowing earlier mobilization and weight-bearing [16,17].

Surgical concepts have substantially improved over the past decades, leading to a significant reduction in complications and infection rates. In the 1990s, bicondylar TPF was associated with infection rates of up to 87.5% [28]. The introduction of staged surgical management, combined with modern fracture-specific approaches and improved implants, has reduced infection rates to approximately 4–5% in recent studies [28,39,40]. Adjunctive strategies such as local antibiotic application and optimized perioperative management have further contributed to these improvements.

Despite these advances, several important unresolved challenges remain. One of the most significant open questions concerns the optimal surgical approach and potential extensions required for complex fracture patterns. Although numerous approaches have been described, there is currently no clear consensus regarding which approach provides the best balance between adequate visualization of the articular surface, safe implant positioning, and minimal soft-tissue complications. Future comparative clinical studies are needed to determine which strategies provide the most reliable outcomes.

Another major challenge is the management of fractures in the elderly with osteoporotic bone and periprosthetic fractures. Demographic changes and increasing life expectancy have led to a growing incidence of fragility fractures and periprosthetic fractures around the knee. In these patients, compromised bone quality frequently limits the ability to achieve sufficiently stable fixation, even with modern implants. Consequently, the development of new fixation strategies and implants specifically designed for osteoporotic bone remains an important area for future research.

## Data Availability

The original contributions presented in this study are included in the article. Further inquiries can be directed to the corresponding author.
